# CD9 protects the sperm from cytotoxic factors in the epididymis as extracellular components

**DOI:** 10.17912/micropub.biology.000950

**Published:** 2023-09-20

**Authors:** Woojin Kang, Kazuki Sugiyama, Daiki Katano, Sae Horiike, Hiromu Morimoto, Ban Sato, Natsuko Kawano, Mitsutoshi Yamada, Mami Miyado, Kenji Miyado

**Affiliations:** 1 Laboratory Animal Resource Center, Transborder Medical Research Center, Faculty of Medicine, University of Tsukuba, Tsukuba, Ibaraki, Japan; 2 Department of Reproductive Biology, National Research Institute for Child Health and Development, Setagaya-ku, Tokyo, Japan; 3 Department of Life Sciences, School of Agriculture, Meiji University, Tama-ku, Kawasaki, Kanagawa, Japan; 4 Department of Bioscience, Graduate School of Life Science, Tokyo University of Agriculture, Setagaya-ku, Tokyo, Japan; 5 Department of Nutritional Science and Food Safety, Faculty of Applied Bioscience, Tokyo University of Agriculture,Setagaya-ku, Tokyo, Japan; 6 Department of Obstetrics and Gynecology, Keio University School of Medicine, Shinjuku-ku, Tokyo, Japan; 7 Department of Food Science and Human Nutrition, Beppu University, Beppu, Oita, Japan; 8 Department of Molecular Endocrinology, National Research Institute for Child Health and Development, Setagaya-ku, Tokyo, Japan; 9 Division of Diversity Research, National Research Institute for Child Health and Development, Setagaya-ku, Tokyo, Japan

## Abstract

The mechanism by which seemingly normal sperm cause infertility is still under debate. Although CD9 is expressed in male reproductive tissues, its role in male fertility remains unclear. To address this, we investigated the role of CD9 in analyzing *Cd9* -deficient ( *Cd9* -KO) male mice. The litter size of *Cd9* -KO males was comparable, regardless of mating experience. When *Cd9* -KO males experienced their first mating chance, a considerable number of neonates died 48 hours after birth. Electron microscopy reveals the presence of CD9 in the epididymal space. Our results suggest that CD9 contributes to male fertility as an extracellular component.

**Figure 1. Possible role of CD9-containing extracellular structures in the epididymal cavity:**
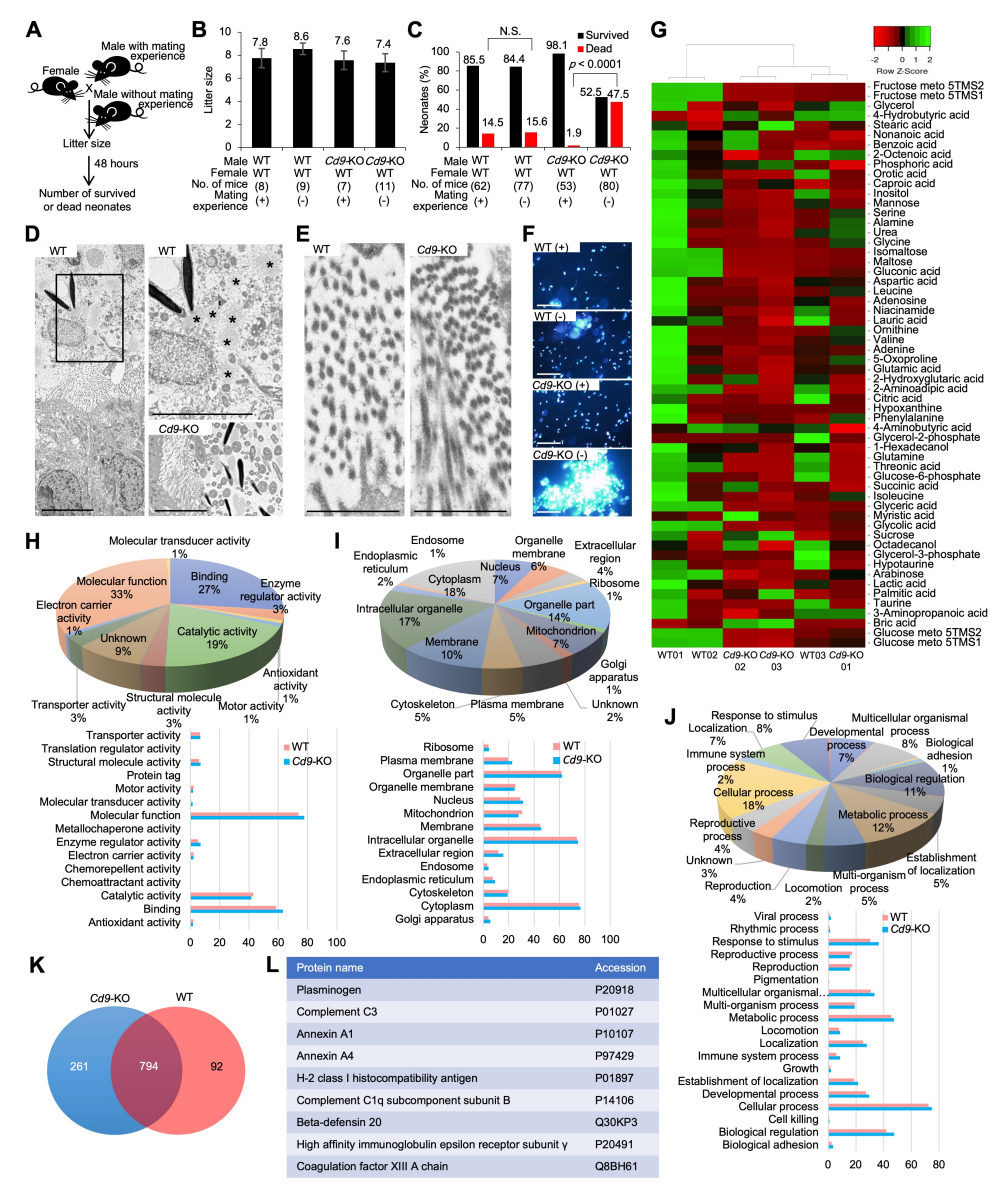
**(A)** Experimental flow. Litter size was examined in *Cd9* -deficient (KO) and wild-type (WT) male mice with or without mating experience. All male mice were mated with WT female mice. The number of dead and survived neonates was examined 48 hours after birth. **(B)** Litter size. No. of mice indicates the number of pairs examined. Values are expressed as mean ± standard error. **(C)** Neonatal death. The percentage of dead and survived neonates was compared among mating combinations. No. of mice indicates the number of neonates examined. The values were analyzed by a chi-squared test. **(D)** Transmission electron microscopic (TEM) images of the epididymal cavity in male mice without mating experience. Scale bars: 5 μm. **(E)** TEM images of microvilli of the epididymal epithelium in male mice without mating experience. Scale bars: 5 μm. **(F)** Microscopic observation of the sperm. The sperm were collected from the epididymis, cultured for 2 hours in a fertilization medium. The sperm nuclei were stained with Hoechst 33342 before observation. WT (+) and *Cd9* -KO (+): male mice with mating experience. WT (-) and *Cd9* -KO (-): male mice without mating experience. Scale bars: 80 μm. **(G)** Metabolomic analysis of the epididymal sperm. Three samples (each sample obtained from 2–3 male mice) were prepared from *Cd9* -KO and WT male mice without mating experience, respectively. **(H–L)** Proteomic analysis of the epididymal sperm collected from male mice without mating experience. **(H)** Gene Ontology (GO) enrichment analysis (GO term: molecular function). **(I)** GO enrichment analysis (GO term: cellular component). **(J)** GO enrichment analysis (GO term: biological process). **(K)** Venn diagram of all proteins detected. **(L)** List of typical proteins detected only in *Cd9* -KO sperm.

## Description

Currently, approximately half of all infertility cases originate in men (Kuroda et al. 2020) , but most of the pathogenic mechanisms of male infertility remain unresolved. The number of people suffering from infertility is likely to increase owing to the trend toward late marriages and the consequent increase in childbearing age. Aging also affects male fertility, and semen volume and sperm motility are reduced (Uchida et al. 2023) . Additionally, the expression of inflammatory cytokines in the testes increases with age (Li et al. 2023) . As childbearing age is expected to increase further in the future, elucidating the effects of aging on male fertility is urgently needed.

In multicellular organisms, cellular components are constantly translocated within cells and secreted as components of extracellular vesicles. Exosomes function as key mediators of intercellular transportation (Tkach and Thery 2016; El-Tanani et al. 2023) . An exosomal component, tetraspanin CD9, regulates sperm–egg fusion in mammals (Miyado et al. 2000) . CD9 is not only localized on the plasma membrane but is also incorporated into several types of microvesicles, typically exosomes (Miyado et al. 2008; Miyado et al. 2019) . Moreover, CD9-containing structures (or “microexosomes”) are released from mouse eggs during their maturation and promote sperm–egg fusion. CD9 is widely expressed in cells, regardless of their membrane fusion ability. Therefore, microexosomes are expected to play important roles in cellular functions beyond membrane fusion. Microexosomes are released from uterine epithelial cells and promote membrane regeneration, resulting in cyclic uterine repair in mice and, presumably, humans (Kawano et al. 2014; Iwai et al. 2019) .

*Cd9* -deficient ( *Cd9* -KO) mice develop senescence-like symptoms in the retina (Iwagawa et al. 2020) and skin regeneration (Takeda et al. 2003; Zhang et al. 2012) . These symptoms are widely observed in *Cd81* / *Cd9* double-deficient (DKO) mice (Takeda et al. 2008) . Although DKO mice could be useful as a model of aging, how senescence caused by the loss of either CD9 or CD81 affects male fertility is unknown. In this study, we explored the roles of CD9 and CD9-containing extracellular structures in male fertility.

To examine litter size, *Cd9* -KO male mice were mated with female wild-type (WT) mice ( Fig. 1A, B ). Male WT control mice were mated with female WT mice. To evaluate the influence of mating experience, male mice were divided into two groups: with or without mating experience ( Fig. 1A –C). Despite no difference in litter size among the groups, the percentage of dead neonates significantly increased in *Cd9* -KO male mice with first mating experience ( *p* < 0.0001).

As sperm maturation is affected by epididymal transit time (Fernandez et al. 2008) , dead neonates may originate from damaged sperm. If the time between sperm production and ejection is too long in male mice without mating experience, sperm may be damaged, especially in the epididymis of *Cd9* -KO mice. To address this, we examined the histological features of the epididymal cavity and sperm. Transmission electron microscopy revealed that the epididymal cavity contained microexosome-like structures in WT male mice but not *Cd9* -KO male mice ( Fig. 1D ). Furthermore, the space surrounding microvilli of the epididymal epithelium was narrower in *Cd9* -KO male mice than in male WT mice ( Fig. 1E ), implying the loss of CD9-containing extracellular structures.

To study the physiological characteristics of epididymal sperm, the sperm were collected from *Cd9* -KO and WT male mice. Both strains of mice were divided into two groups with and without mating experience, *Cd9-* KO (+) and WT (+), and *Cd9* -KO (-) and WT (-) ( Fig. 1F ). After the sperm were incubated in a fertilization medium, their nuclei were stained with Hoechst 33342. Among mating combinations, hundreds of the sperm from *Cd9* -KO (-) mice clustered in the absence of eggs, implying that their sperm membrane may be damaged.

To investigate the occurrence of problems in sperm from *Cd9* -KO male mice, the quantity of metabolites was compared between the sperm from *Cd9* -KO and WT male mice ( Fig. 1G ). Fifty-eight metabolites were detected, but their levels were comparable in the sperm of *Cd9* -KO and WT male mice.

Proteomic analysis was performed to explore the differences in sperm between *Cd9* -KO and WT male mice. Gene ontology (GO) annotation revealed the three most relevant GO terms (in molecular function, cellular component, and biological process) ( Fig. 1H –L), but no difference was observed in these three GO terms. The Venn diagram revealed that 794 proteins overlapped; 261 proteins were detected only in the sperm of *Cd9* -KO male mice and 92 proteins were detected only in the sperm of WT male mice ( Fig. 1K ). Nine typical proteins detected only in the sperm of *Cd9* -KO male mice were related to inflammatory and innate immune responses ( Fig. 1L ).

We here showed that extracellular CD9-containing structures exist in the epididymal cavity. Correspondingly, the sperm from *Cd9* -KO male mice, especially without mating experience, were damaged inside the epididymis. Thus, we propose that CD9 protects epididymal sperm from environmental factors, including mechanical stress, immune responses, and subsequent cholesterol loss from the sperm membrane. Epididymal function contributes to sperm quality (Gao et al. 2023) . The testes have normal spermatogenesis, but apoptotic activity increases in the epididymis of patients and mice with obstructive azoospermia, resulting in reduced sperm quality. Because conventional cryopreservation methods induce chemical and mechanical damage to sperm membranes (Sicchieri et al. 2021) , epididymal sperm may be exposed to similar mechanical damage. The epididymis functions as a transition route for post-testicular sperm maturation and storage, and faces immunological responses toward the sperm. Up to 15% of male patients with infertility experience immune responses due to pathological infections and autoimmune responses affecting the male reproductive organs, including the epididymis and prostate. The epididymis has attracted interest because of its relationship with immune responses (Voisin et al. 2019) . Sperm carry unique membrane proteins that are not recognized as self-antigens. These proteins are potential targets of immune responses with the risk of causing the production of autoantibodies and, consequently, male infertility. Epididymal immunity is based on a finely tuned mechanism involving immune responses to pathogens and sperm tolerance. Our results contribute to the understanding of the roles of proteins and structures in the epididymal space and male fertility.

## Methods

***Cd9* -deficient ( *Cd9* -KO) mice**

Epididymis and epididymal sperm were isolated from 8-12-week-old *Cd9* -KO male mice. C57BL/6J male and female mice were purchased from Japan SLC Inc. (Shizuoka, Japan). All mice were housed under specific pathogen-free conditions. Food and water were provided ad libitum. The procedures for performing the animal experiments were approved by the Institutional Animal Care and Use Committee of the National Research Institute for Child Health and Development (approval letter number: A2004-004).

**Counting the number of dead and survived neonates**

The number of neonates that died by cannibalization and abandonment 48 hours (h) after birth was counted. The number of neonates that remained after 48 h was counted as the surviving neonates.

**Transmission electron microscopy**

The epididymis was isolated from 8–12-week-old male mice and fixed in 50 mM sodium cacodylate, pH 7.2, containing 2.5% glutaraldehyde and 2% sucrose, for 2 h at 4 °C, as described previously (Kang et al. 2020) . The fixed epididymis was then rinsed three times and post-fixed in a fixative containing 2% osmium tetroxide for 2 h at 4 °C. The specimens were dehydrated in ethanol, immersed twice in propylene oxide for 15 min each, and embedded in Epon. Ultrathin sections were prepared using an ultramicrotome (Reichert Ultracuts; Leica AG, Vienna, Austria) and stained with uranyl acetate and lead citrate. Sections were observed under a transmission electron microscope (H-7000; Hitachi, Tokyo, Japan).

**Microscopic observation of the sperm**

Sperm collected from the epididymis of 8-12-week-old male mice were induced to capacitate by incubating in a fertilization medium (Toyoda-Yokoyama-Hoshi medium; TYH medium) (Toyoda et al. 1971) for 2 h in an atmosphere of 5% CO _2_ in air at 37°C. The sperm nuclei were then stained with Hoechst 33342 at the final concentration of 5 μg/mL, and were observed under a IX71 microscope (Olympus, Tokyo, Japan).

**Metabolomic analysis**

To prevent contamination with epididymal fluids, epididymal sperm were washed with Hank’s balanced salt solution (Thermo Scientific, Rockford, IL, USA) and immediately frozen in liquid nitrogen. As described previously (Kang et al. 2020) , each sample (2 ´ 10 ^7^ sperm, approximately 20 mg) was placed in methanol (75–80% final concentration) and homogenized using zirconia beads. The homogenates were centrifuged at 18,000 ´ *g* for 5 min and the supernatants were purified on pretreated spin columns (MonoSpin C18; GL Sciences, Saitama, Japan). The samples (each sample obtained from 2–3 male mice) were analyzed using an Agilent 7890A gas chromatography (GC) system coupled to an Agilent 5975C inert XL MSD with a triple-axis mass detector, Agilent 7693 Series Autosampler, and DB-5 capillary column (Agilent Technologies, Santa Clara, CA, USA).

**Shotgun proteomics**

The sperm were extracted in 50 μL of lysis buffer (8 M urea and 0.2 M ammonium bicarbonate) and sonicated. Each lysate was digested with trypsin and labeled with iTRAQ 4-plex reagent (Merk, Darmstadt, Germany) according to the manufacturer’s instructions. An Orbitrap Q Exactive Plus spectrometer (Thermo Fisher Scientific, Waltham, MA, USA) and EASY-nLC 1200 chromatograph (Thermo Fisher Scientific) were used to generate the shotgun proteomic data. Liquid chromatography-tandem mass spectrometry (LC-MS/MS) and database searches were performed using APRO Science (Tokyo, Japan).

**Statistical analysis**

Statistical significance was analyzed using Student’s t-test and chi-squared test. A result with *p* < 0.05 was considered significant. Values are expressed as the mean ± standard error.

## Reagents

C57BL/6J and *Cd9* -KO mice
